# Laser‐Induced Graphene Enabled Additive Manufacturing of Multifunctional 3D Architectures with Freeform Structures

**DOI:** 10.1002/advs.202204990

**Published:** 2022-11-27

**Authors:** Fu Liu, Yan Gao, Guantao Wang, Dan Wang, Yanan Wang, Meihong He, Xilun Ding, Haibin Duan, Sida Luo

**Affiliations:** ^1^ School of Mechanical Engineering & Automation Beihang University No. 37 Xueyuan Road Beijing 100191 China; ^2^ School of Automation Science and Electrical Engineering Beihang University No. 37 Xueyuan Road Beijing 100191 China

**Keywords:** 3D graphene macrostructures, 3D printing, flexible electronics, laser‐induced graphene, multifunctional structures

## Abstract

3D printing has become an important strategy for constructing graphene smart structures with arbitrary shapes and complexities. Compared with graphene oxide ink/gel/resin based manners, laser‐induced graphene (LIG) is unique for facile and scalable assembly of 1D and 2D structures but still faces size and shape obstacles for constructing 3D macrostructures. In this work, a brand‐new LIG based additive manufacturing (LIG‐AM) protocol is developed to form bulk 3D graphene with freeform structures without introducing extra binders, templates, and catalysts. On the basis of selective laser sintering, LIG‐AM creatively irradiates polyimide (PI) powder‐bed for triggering both particle‐sintering and graphene‐converting processes layer‐by‐layer, which is unique for assembling varied types of graphene architectures including identical‐section, variable‐section, and graphene/PI hybrid structures. In addition to exploring combined graphitizing and fusing discipline, processing efficiency and assembling resolution of LIG‐AM are also balanceable through synergistic control of lasing power and powder‐feeding thickness. By further studying various process dependent properties, a LIG‐AM enabled aircraft‐wing section model is finally printed to comprehensively demonstrate its shiftable process, hybridizable structure, and multifunctional performance including force‐sensing, anti‐icing/deicing, and microwave shielding and absorption.

## Graphical abstract

In this work, a laser‐induced graphene based additive manufacturing (LIG‐AM) is developed to print bulk 3D graphene with freeform shapes such as identical‐section, variable‐section, and graphene/PI hybrid structures. By investigating particle‐sintering and graphene‐converting dual‐mechanism as well as process dependent properties, an aircraft‐wing section model is finally printed to demonstrate its shiftable process, hybridizable structure, and multifunctional performance.

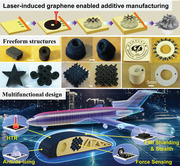

## 1 Introduction

Nowadays, 3D printing has become the most powerful technology for rapid designing, prototyping, and manufacturing structures, components, and products with multidimensional geometries and random complexities in almost all industries.^[^ ^1^, ^2^ ^]^ In addition to fulfilling freeform shapes with requisite mechanical properties, structural functionalization is also inevitable to meet the future need of 3D‐printed objects with single or multiple advanced or smart performances through multi‐material‐printing,^[^ ^3^ ^]^ surface‐modifying,^[^ ^4^ ^]^ topological‐structuring,^[^ ^5^ ^]^ and nanomaterial‐integrating^[^ ^6^ ^]^ strategies. As one of the most promising nanotechnologies, graphene has attracted tremendous attention for assembling smart devices and structures with various geometries from 1D to 3D based on its hexagonal‐latticed molecules,^[^ ^7^ ^]^ embeddable/connectible/modifiable 2D nanostructures,^[^ ^8^, ^9^, ^10^ ^]^ as well as extraordinary electrical, mechanical, thermal, and optical properties.^[^ ^11^, ^12^, ^13^, ^14^ ^]^ For example, graphene‐enabled 1D filaments, fibers and yarns,^[^ ^15^, ^16^, ^17^ ^]^ 2D thin films, papers and fabrics,^[^ ^18^, ^19^, ^20^ ^]^ and 3D foams, hydrogels, and millispheres^[^ ^21^, ^22^, ^23^ ^]^ have been explored extensively by endowing the assembled macrostructure with free‐standing skeletons,^[^ ^24^ ^]^ hierarchical microporosities,^[^ ^25^ ^]^ ultralow density,^[^ ^26^ ^]^ and interconnected high‐conductive networks^[^ ^24^ ^]^ for enabling critical components in wearable electronics,^[^ ^20^ ^]^ thin‐film batteries,^[^ ^8^ ^]^ artificial skins/muscles,^[^ ^27^, ^28^ ^]^ biomimetic robots,^[^ ^29^ ^]^ self‐sensing composites,^[^ ^30^ ^]^ etc.

To manufacture macroscopic graphene ensembles, two processing strategies have been applied majorly including bulk formation and layer‐by‐layer assembly. The former relies on non‐printing processes to either synthesize graphene on 3D porous metal templates or hydrothermally treat graphene oxide (GO) dispersion to form bulk foam structures. For example, by using chemical vapor deposition (CVD) protocol, Cheng et al.^[^ ^21^ ^]^ and Shi et al.^[^ ^26^ ^]^ respectively grew graphene on macroporous nickel and seashell templates to form freestanding foam‐like structures with the size of 20 × 20 × 2 mm^3^ after the etching process. Without introducing issues of high temperature and rigorous atmosphere control, Xu et al.^[^ ^31^ ^]^ and Yu et al.^[^ ^32^ ^]^ respectively applied hydrothermal and directional‐freezing approaches to cast and reduce GO suspension into self‐assembled graphene hydrogels or aerogels with the cylinder‐shaped volume around 9 cm^3^. Although bulk formation strategy could reach centimeter‐scaled architecture, it is unable to achieve arbitrary shapes due to the lack of programmable working path. As an advanced alternative, layer‐by‐layer assembly relies on computer‐aided process to create 3D graphene monoliths or patterns through different additive manufacturing manners including inkjet printing,^[^ ^33^ ^]^ extrusion printing,^[^ ^27^ ^]^ stereolithography,^[^ ^34^ ^]^ kirigami patterning,^[^ ^35^ ^]^ etc. With the feature size of micron scales, for example, Hensleigh et al.^[^ ^36^ ^]^ and Jiang et al.^[^ ^33^ ^]^ respectively applied photocurable GO resin and GO sol‐gel ink to print self‐standing 3D graphene structures with programmable microlattices. The layer‐by‐layer based methods could certainly promote the complexity and designability of structures; nevertheless, most of them rely on GO based inks, gels and resins as raw printing materials, still facing two major drawbacks. Firstly, the GO wet chemistry requires complex processing steps from suspension preparation, layer‐by‐layer stacking, freeze‐drying, to various chemical/physical treatments that dramatically prolong the working period and limit the size scalability.^[^ ^37^ ^]^ Correspondingly, the quality control in each step is tricky for tackling ink stability, printing rheology, GO reduction, structural shrinkage, etc. These further intensify the molding difficulty and part‐to‐part variations.^[^ ^38^ ^]^ Thus, a new 3D printing strategy toward swift assembly, freeform structuring, and ease of quality control is highly anticipated for construction of graphene macrostructures.

As a newly‐developed technique, laser‐induced graphene (LIG) has dramatically promoted the possibility of graphene assembly by introducing multiple unique characteristics including direct laser scribing, one‐step graphitic conversion, various compatible precursors, and customizable working routes.^[^ ^39^ ^]^ By avoiding complexities of ink and catalyst preparations in traditional liquid‐assembling and CVD methods, LIG indeed has reflected its facile, low‐cost and scalable advantages for fabricating various single‐layered structures including graphene fibers,^[^ ^40^ ^]^ thin films,^[^ ^41^ ^]^ papers,^[^ ^42^ ^]^ and surface patterns,^[^ ^43^ ^]^ which have become core elements in various novel structures and devices, e.g., functional composites,^[^ ^44^ ^]^ supercapacitors,^[^ ^41^ ^]^ heaters,^[^ ^45^ ^]^ environmental,^[^ ^46^ ^]^ and biological devices.^[^ ^47^ ^]^ For assembly of macroscopic 3D objects, although several initial trials have emerged, LIG has still faced clear obstacles. For example, Luong et al.^[^ ^48^ ^]^ developed automated laminated object manufacturing (LOM) to fabricate centimeter‐sized graphene monoliths by repeatedly converting, fusing and milling Kapton films into stacked LIG layers with different shapes. With similar strategy, our group applied hot press to one‐step molding multiple pre‐impregnated LIG/epoxy papers to form 3D‐lamiated graphene composites with the size as large as 20 × 20 × 0.2 cm^3^.^[^ ^49^ ^]^ Although macroscopic dimensions are achievable, the above works require specific binding, milling, and/or 3D‐molding processes which have restricted the bulk formation of graphene with simple shapes. By raising a binder‐free strategy, Sha et al.^[^ ^50^ ^]^ applied sucrose and nickel particle mixtures as carbon source and catalyst to grow graphene layer‐by‐layer on the metal template. Although it is able to construct 1 × 1 × 0.8 cm^3^ sized foams, the post template‐etching treatment inevitably causes considerable volume shrinkage that confines the shaping accuracy. Other strategies such as LIG processed on preprinted 3D plastics,^[^ ^51^ ^]^ composites,^[^ ^52^ ^]^ and kirigami/origami patterns^[^ ^44^ ^]^ are also not able to construct freeform graphene structures. Thus, a novel LIG process toward the ease of freeform manufacturing of graphene 3D macrostructures is still not appeared.

Following this line of thought, in this work we developed a brand‐new LIG based additive manufacturing (LIG‐AM) protocol to form bulk graphene structures with freeform geometric shapes. Without introducing extra binder, template and catalyst, polyimide (PI) powder was applied as the only raw materials for both particle‐sintering and graphene‐converting purposes to construct 3D solids. Based on computer‐aided laser‐irradiation scribing to selectively process each layer with individually programmed slicing pattern, LIG‐AM is able to analogize the commercial selective laser sintering (SLS) technology to form varied types of bulk architectures including identical‐section, variable‐section, and even unique graphene/PI hybrid structures with random shape complexities. In addition to investigate the combined graphitizing and fusing discipline toward macroscopic assembly, systematic work has also been done to balance the processing efficiency and assembling resolution that are controlled interactively by laser power and powder‐feeding thickness. By further studying the tunable electrical, mechanical, piezoresistive, liquid‐sensing, and joule‐heating properties as well as resin‐integration reinforced strength and stretchability, a LIG‐AM enabled aircraft‐wing section model was specifically designed to comprehensively demonstrate its shiftable printing strategy, hybridizable structure, and multifunctional performance including multidirectional force‐sensing, anti‐icing/deicing, and microwave shielding and absorption.

## 2 Results and Discussion

### 2.1 Freeform Manufacturing and Processing Mechanism

**Figure 1:**
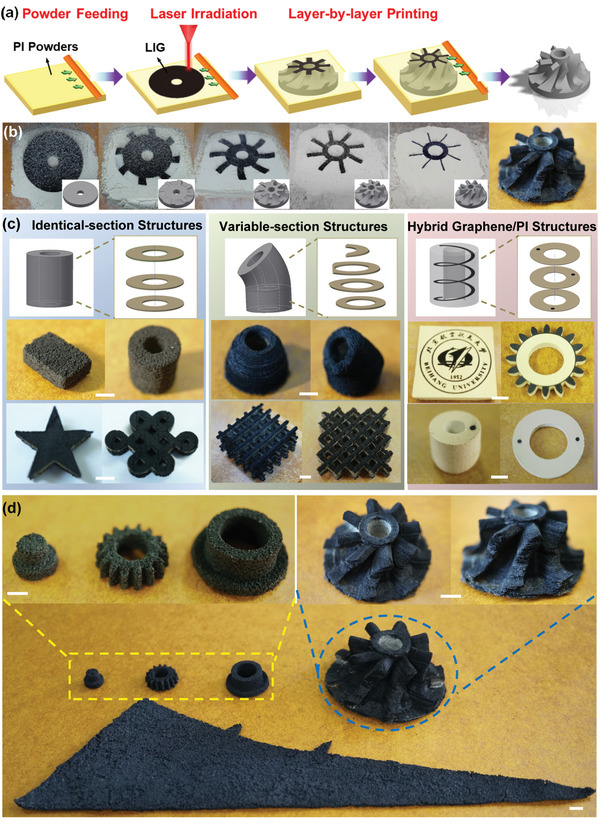
Concept and demonstration of LIG‐AM process. a) Schematic illustration and b) step‐by‐step process for printing a whole graphene turbine. Demonstrations of LIG‐AM enabled 3D objects with c) identical‐section structures, variable‐section structures, hybrid graphene/PI structures, and d) multiscale dimensions. (Scale = 5 mm)

Inspired by the commercial SLS manufacturing to print 3D metallic, ceramic, and polymeric objects through layer‐by‐layer fusion of powders, the idea of using compatible powder‐based LIG raw materials is the key for constructing graphene macrostructures with freeform geometric shapes. In this work, commercially available thermoplastic PI powders with the size of ≈10 µm was selected as the ideal raw materials by competing other LIG‐compatible powders considering irradiation energy, degree of graphitization, and shape accuracy (Figure S1, Supporting Information). By serving both particle‐sintering and graphene‐converting functions, the proposed LIG‐AM protocol is indeed feasible for additive manufacturing of various graphene structures. As schematically depicted in **Figure** **1**a, a computer‐controlled CO_2_ laser system serves as the power source to selectively solidify and graphitize the very top layer of powder bed with a preprogrammed slicing pattern before feeding a new layer to finally construct a printed 3D object. To specify, a thin layer of PI powder is firstly fed onto the working plate using a spacer to control the height of powder bed. Then, the laser irradiates and scribes the powder bed to selectively sinter and convert PI powder into a continuous graphene structure with specific surface pattern. Subsequent to the first LIG layer formation, a new powder layer is fed on top and followed by another irradiation process. The feeding and irradiating steps then repeat multiple times until processing all layers to present the entire structure after removing unprocessed powders.

By taking best advantage of SLS along with simultaneous laser‐induced graphene process, the LIG‐AM is able to construct macroscopic graphene with high level of complexity. Figure 1b and Video S1 (Supporting Information) demonstrate the process of LIG‐AM to print a whole graphene turbine. A series of pictures respectively show the appearance of powder‐bed after processing the 1st, 20th, 50th, 100th, and 200th layer. In every layer, the surface of ash‐colored powder bed partially turns black with specific shape pattern, indicating the graphene‐conversion process by following the irradiating route based on the slicing data. After extracting the printed object out of powder bed, the graphene turbine is displayed with a round‐disc base (5 cm in diameter), a hollow shaft (2.3 cm height), and 8 twisted blades, as shown in Figure S2 in the Supporting Information. According to the freeform processing, we further demonstrated the capability of LIG‐AM for constructing diverse graphene architectures, which have been categorized into identical‐section, variable‐section, and hybrid graphene/PI structures. As displayed in Figure 1c, the identical‐section structure (left column) applies the same slicing area to accumulate its height. For example, centimeter‐scaled cuboid block, hollow cylinder, pentagram prism, and Chinese knot structures can be printed. The variable‐section structure (middle column) applies gradual‐changed or interchanged slicing area to achieve more special shapes. For example, two peculiar tubes were printed including one with a straight channel but a tapering shell and another one with a curved channel. Similarly, two lattice structures were printed with different types of crossbar topologies. The hybrid structure (right column) is rather unique by shifting low and high‐leveled lasing energy to selectively sinter or graphitize PI. Thus, the graphene structure can merge with PI matrix by either presenting on top, interfacially connected or internally inhabited. For example, a graphitized Beihang‐logo pattern was printed on top of a PI block; a PI gear wheel was printed with 16 graphene teeth; graphene channels were printed inside a tube and a ring component with only inlet and outlet spots appeared on surface. Figure S3 in the Supporting Information demonstrates the electroconductibility of the internal channels. Lastly, Figure 1d represents the feasibility of LIG‐AM for manufacturing multiscale structures from a 9 mm sized component to a 21.3 cm long airplane wing.

**Figure 2:**
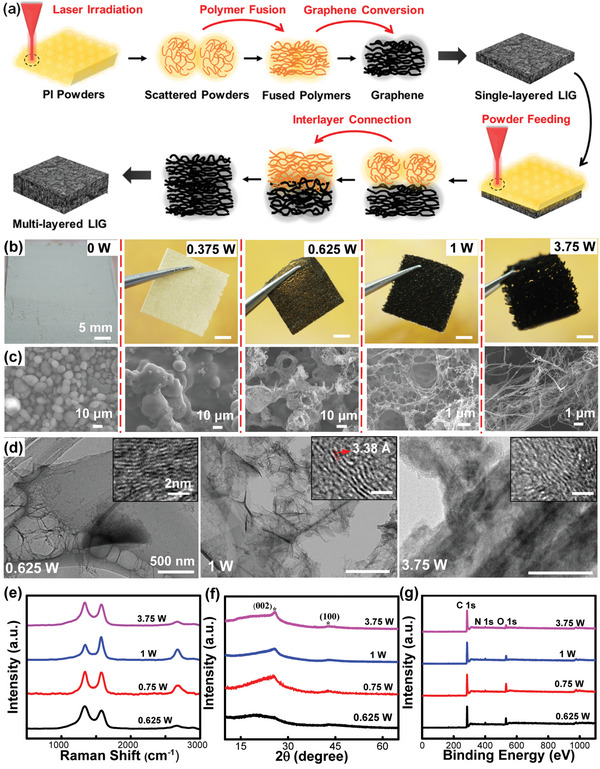
Processing mechanism and structural characterization. a) Schematic diagram of LIG‐AM to process multi‐layered structures. b) Photographs, c) SEM images, d) TEM images, e) Raman spectra, f) XRD patterns, and g) XPS survey spectra of LIG‐AM enabled structures processed by different irradiation conditions.

To understand the mechanism of LIG‐AM, **Figure** **2**a schematically explains the process to form a multi‐layered graphene structure involving polymer flow, graphene conversion, and interlayer connection. Upon laser irradiation, the intra‐layered PI powders are subjected to an ultrafast thermal cycle that the heated temperature first unfreezes polymer chains with certain flowability to introduce interfacial connections among neighboring powders. The fused polymers are further carbonized and graphitized when the continuously elevated temperature decomposes and regroups certain chemical bonds into crystalized graphene structures.^[^ ^41^, ^53^ ^]^ Thus, by applying the thermal cycle to process the whole powder layer, a continuous graphene film can be formed by converting entangled polymer network to porous graphene structure. After feeding a new powder layer, the same irradiation treatment induces the formation of another graphene layer, and simultaneously, the melted polymers infiltrate inside the underlying graphene network for introducing strong interlayer connections before graphitization process.

Based on mechanism analysis, it is reasonable to believe that the level of irradiation energy is the key to control the dual functionality of PI for both particle sintering and graphene conversion. Figure S4 in the Supporting Information first characterizes basic properties of PI powders, including size distribution (10±2 µm), glass transition temperature (≈263 °C), and three‐stage weight loss at ≈200 °C (water evaporation), ≈500 °C (oxygen and nitrogen outgassing), and ≈700 °C (carbonization), suggesting both melting‐ and LIG‐processibilities. To experimentally support this, Figure 2b,c reveals macroscopic appearance and microscopic morphology of representative single‐layered samples processed under varied laser powers. Compared with neat powder bed with clear boundary of particles, low laser power (e.g., 0.375 W) is able to fuse particles together and form a PI film when the heated temperature is high enough to partially melt PI. With moderate power (e.g., 0.625 W), the processed film starts to show black appearance with coexistence of uncarbonized PI, indicating the threshold power of carbonization. Meanwhile, the fused bump‐like microstructure starts to show some pores, confirming the decomposition of chemical groups with gas release. When laser power ranges from 0.75 to 2.5 W (e.g., 1 W), the film surface becomes totally black and its microscopy shows iconic LIG morphology with uniform pore distribution. Specifically, the foam‐like structure is the result of laser photothermal reaction to rearrange the decomposed C—O, C=O, or —C─N bonds and ablate sp^3^ C—C to form long‐range ordered graphene layers.^[^ ^54^ ^]^ By exceeding 3 W (e.g., 3.75 W), the macroscopic LIG structure is no longer mechanically stable with considerable voids observable in the fibrous microstructure, indicating excessive ablation effect to destroy the LIG framework. More detailed scanning electron microscopy (SEM) images are displayed in Figure S5 in the Supporting Information.

In addition to optical and SEM analyses, a series of structural characterizations were further explored to support the process dependent graphene conversion. In contrast to 0.625 and 3.75 W processed samples, transmission electron microscopy (TEM) images in Figure 2d first unveil the most prominent structure of few‐layered graphene with ripple‐like wrinkles and a lattice space of 0.34 nm.^[^ ^41^ ^]^ Raman spectroscopy in Figure 2e and Figure S6 (Supporting Information) further summarizes the dynamic change of characteristic peaks of graphitic crystals. As the increase of laser power from 0.375 to 3.5 W, both the intensity ratio of G to D (*I*_G_/*I*_D_) and 2D to G (*I*_2D_/*I*_G_) peaks follow similar trend that the sample processed around 1 W shows both the highest *I*_G_/*I*_D_ (1.37) and *I*_2D_/*I*_G_ (0.41), indicating the best graphene quality with the fewest number of layers.^[^ ^55^ ^]^ Additionally, X‐ray diffraction (XRD) in Figure 2f shows the processing power reaching at least 0.75 W to observe the prominent peak of (002) graphitic crystal phase at 2‐theta angle of 26°, indicating a high degree of graphitization.^[^ ^56^ ^]^ Similarly, X‐ray photoelectron spectroscopy (XPS) in Figure 2g discovers the major element carbon (≈284 eV) in the aryl group and oxygen (≈532 eV) in the ketone bond and nitrogen (≈402 eV) in various samples. By increasing the laser power, the C/O ratio is continuously enhanced from 2.9 for 0.375 W processed PI film to 15.1 for 3.75 W processed graphitic film. Based on the above results, high laser power induces high degree of carbonization and graphitization, but a proper processing window ranged from 0.75 to 2.5 W guarantees the printed object with better graphene quality and mechanical stability.

### 2.2 Control of Processing Efficiency and Assembling Resolution

**Figure 3:**
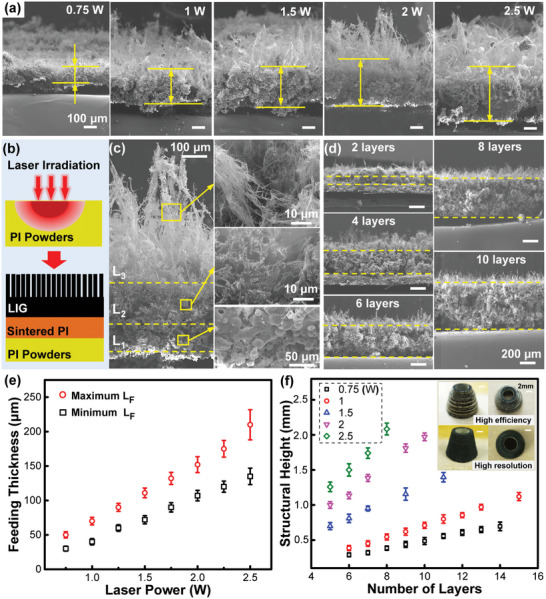
Processing efficiency and assembling resolution. a) Thickness of single‐layered LIG films processed by different laser powers. b) Schematic diagram and c) SEM images of single‐layered LIG with three sublayers. d) Cross‐sectional structures of LIG with increased layer numbers. e) Maximum and minimum of powder‐feeding thickness for every laser processing condition. f) Relationships between structural height and number of layers for every laser processing condition.

By determining the processing window, laser power is the critical factor to control the quality of graphene because of the variable irradiation energy absorbed by the PI source. Following this clue, it is reasonable to deduce the possibility for controlling and balancing the efficiency and resolution of LIG‐AM for assembling 3D architectures by varying the energy input. To prove this idea, **Figure** **3**a first shows cross‐sectional SEM images of single‐layered samples by irradiating different power‐leveled lasers on a thick powder bed. Overall, the thickness of LIG film is enlarged continuously from ≈125 to ≈345 µm as the increase of laser power from 0.75 to 2.5 W. The monotonic trend can be explained by the heat transfer theory that higher‐leveled laser could radiate and transport more energy to fuse and graphitize deeper PI powders to form a thicker film. By further understanding the discipline of energy dissipation along the thickness direction to possibly introduce temperature gradient, the processed film could form several sublayers as depicted in Figure 3b. SEM images of Figure 3c indeed uncover a representative single‐layered film with a typical three‐sublayer structure. The bottom layer (*L*_1_) shows the fused PI structure, indicating that the transported energy is not high enough for graphitization. Accordingly, a dense LIG layer (*L*_2_) appears right above the PI layer indicating a proper energy for graphene conversion. It is also observed that fiber‐like structures are protruded out of the LIG surface to form a rather loose layer (*L*_3_), which is explained by the quick liberation of pyrolysis gases during the photothermal reaction to distort the LIG structure.^[^ ^57^ ^]^ According to soft nature of *L*_3_, it will merge with the new feeding layer to receive another irradiation treatment (Figure S7, Supporting Information).

Thus, to construct full graphene objects through multi‐layer processing, the thickness of every powder‐feeding layer (*L*_F_) should be critically controlled. Intuitively, *L*_F_ should be smaller than *L*_2_ to ensure all new powders to be converted to graphene; otherwise, *L*_1_ and even unfused PI would appear to destroy the structural integrity. Along with the maximum thickness, the feeding layer *L*_F_ also cannot be too small for causing too much thermal impact. Figure S8 in the Supporting Information compares the 1 W processed LIG structures by varying *L*_F_ from 10 to 90 µm. Clearly, only when *L*_F_ lies between ≈40 and ≈70 µm, uniform multi‐layered LIG can be formed; when *L*_F_ < 40 µm, LIG shows both macroscopic and microscopic defects indicating strong ablation effect; when *L*_F_ > 80 µm, clear delamination has been observed indicating the loss of interfacial bonding. Thus, by applying 50 µm as the proper *L*_F_, Figure 3d further reveals its cross‐sectional microscopy when stacked from 2 to 10 layers. Clearly, a uniform and integral LIG is continuously constructing with the accumulation of structural height from ≈140 to ≈700 µm. By finally determining all maximum and minimum thicknesses, Figure 3e summarizes the appropriate range of powder‐feeding thickness *L*_F_ for every processible condition of laser. Obviously, weaker/stronger laser power is compatible with thinner/thicker *L*_F_, indicating lower/higher processing efficiency but better/worse assembling resolution. To further support this, Figure 3f maps the relationship between structural height and number of layers for all laser conditions, once again proving the augmented printing efficiency (layer height) under higher powered processing. As a specific demonstration as shown in the inset pictures, a 3D tube‐like component with the base diameter of 15 mm and height of 12 mm was constructed by using two printing strategies. The first one selected fast process by using 2.5 W laser and 200 µm *L*_F_ while the second one chose precise process by using 1 W laser and 50 µm *L*_F_. Although the second one took longer time (4 h and 240 layers vs 1 h and 60 layers), it shows a much smoother surface with better graphitized structure.

### 2.3 Multifunctional Properties and Applications

**Figure 4:**
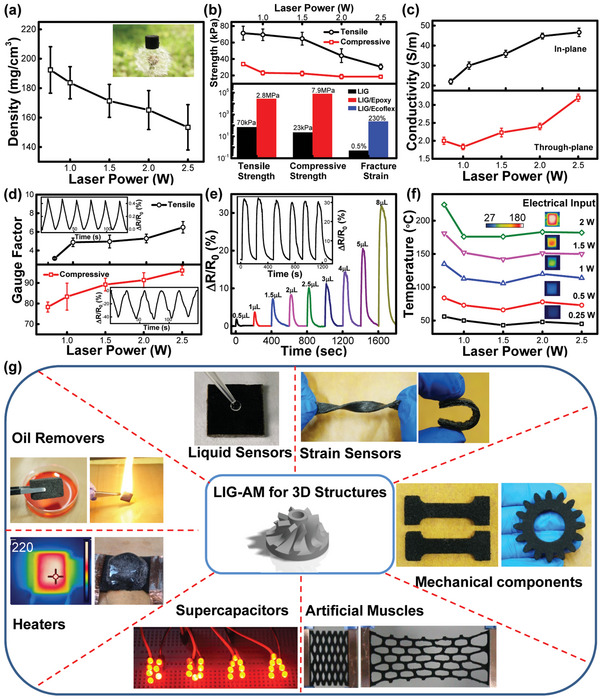
Multifunctional properties and potential applications. Process dependent properties of LIG‐AM enabled structures with tunable a) density, b) mechanical strength, c) electric conductivity, d) piezo‐sensitivity, e) liquid‐sensing capability, and f) Joule‐heating. g) Multifunctional applications of LIG‐AM enabled 3D structures.

By understanding the printing discipline for constructing freeform structures, processing dependent properties of LIG‐AM enabled 3D graphene were further investigated. By using a high‐precision balance for density determination, **Figure** **4**a first unveils the low‐density nature of LIG‐AM structures, descending from 192±15 to 153±6 mg cm^−3^ as the increase of processing power from 0.75 to 2.5 W. Along with the ability to stand on dandelion seeds (inset picture and Figure S9 in the Supporting Information), the monotonic trend further supports the evolved microstructure from mesh‐like foam to fiber‐like network caused by simultaneous modulation of graphitization and ablation (Figure S5, Supporting Information). The structural and chemical evolutions then accordingly determine their mechanical and electrical properties. Specifically, Figure 4b respectively shows the tunable tensile strength (71±8 to 30±3 kPa) along the in‐plane direction and compressive strength (33±2 to 18±1 kPa) along the through‐plane direction of LIG‐AM structures processed under various lasers from 0.75 to 2.5 W. The anisotropic mechanical properties were further evaluated and discussed in Figure S10 in the Supporting Information. Attributed to the porous characteristics, the mechanics of pristine structures can be further reinforced through resin infiltration.^[^ ^21^ ^]^ The inset bar charts compare the LIG/epoxy composites with improved tensile (2.8±0.3 MPa) and compressive strength (7.9±0.5 MPa) and LIG/Ecoflex composites with improved fracture strain (230%). Similarly, LIG‐AM structures reflect an ascending trend on both of their in‐plane (22±1.3 to 46±2.1 S m^−1^) and through‐plane electric conductivity (2±0.1 to 3.2±0.12 S m^−1^) as the increase of processing power (Figure 4c), confirming again the enhanced degree of graphitization. By applying coupled electromechanical measurements, LIG‐AM structures possess great piezoresistive properties according to the quantified gauge factors (Figure 4d). The inset pictures show the real‐time resistance change (Δ*R*/*R*_0_) when subjected to cycled deformations along both tensile and compression directions, exhibiting the linear strain‐induced resistance change with high reproducibility. As the increase of laser power, the increasing trend of both tensile‐deformation determined (3.1±0.1 to 6.4±0.6) and compressive‐deformation determined gauge factor (78±2.4 to 96±2.3) indicates that higher power processed LIG‐AM structures are more sensitive to external disruptions, which coincides with previous finding that high irradiation could induce large numbers of microfissures/cracks in the conducting network.^[^ ^58^ ^]^ In addition to deformation sensing, the porous networks also endows the graphene structures with liquid‐sensing capability.^[^ ^59^ ^]^ Figure 4e shows the 1 W processed LIG‐AM structure for accurately detecting different amount of acetone from 0.5 µL (maximum Δ*R*/*R*_0_ = 1.8%) to 8 µL (maximum Δ*R*/*R*_0_ = 30.5%). The inset plot reveals the resistance‐change cycles with processes of fast rise and gradual recovery, relating to dynamic change of liquid rinsing and evaporation. By virtue of electrothermal characteristics, Figure 4f further demonstrates the joule‐heating performance by electrically powering different LIG‐AM heaters from 0.25 to 2 W to produce thermal energy with the equilibrious heating temperature from 50±6 to 188±20 °C. Under various laser processing conditions, the heaters show similar heating behaviors, suggesting that electrical input power is the dominant factor to control the joule‐heating performance.^[^ ^45^ ^]^ Other functional properties, including hydrophobicity, oil absorption, supercapacitance, and microwave shielding and absorption, were also evaluated and discussed in Figures S11 and S12 in the Supporting Information.

Attributed to freeform 3D construction, selective graphene formation, as well as tunable multifunctionality, the proposed LIG‐AM could become a rather powerful 3D printing protocol for designing, developing and manufacturing various structures, components, and devices for meeting the need of multi‐scenario applications. Figure 4g, Figure S11, Videos S2 and S3 (Supporting Information) demonstrate a series of LIG‐AM enabled smart devices/structures including sensors, heaters, supercapacitors, oil removers, mechanical components, and artificial muscles.

**Figure 5:**
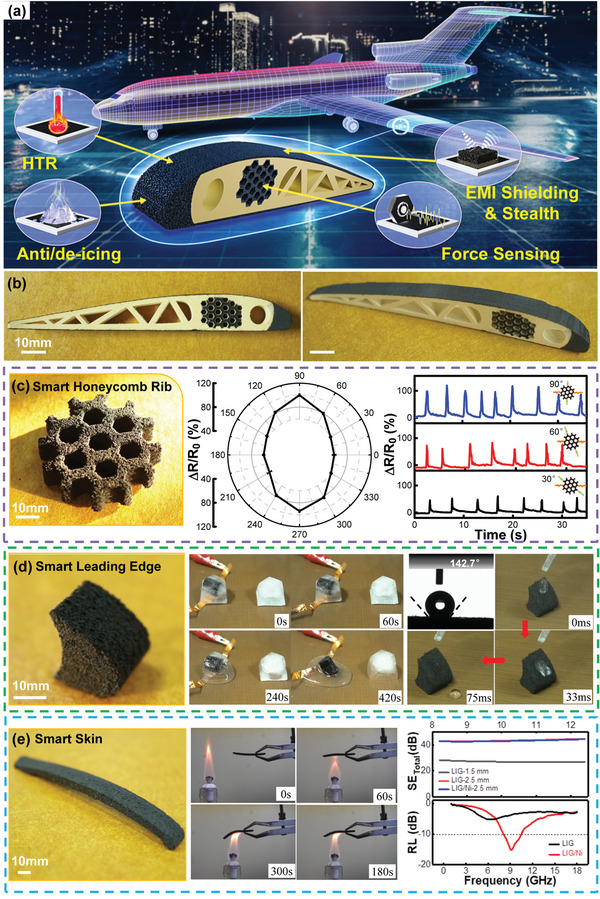
Aerospace application. a) Concept and b) photographs of LIG‐AM printed aircraft‐wing section model. c) Smart honeycomb rib for multidirectional force‐sensing. d) Smart leading edge for anti‐icing/deicing. e) Smart skin for HTR, EMI shielding and stealth.

To demonstrate a specific example for fully exhibiting various unique advantages of LIG‐AM, an aircraft‐wing section model with integrated smart graphene structures was designed, printed and tested. As displayed in **Figure** **5**a,b, the whole model was constructed by LIG‐AM using hybrid printing strategy to shift between low and high power levels to selectively form sintered PI regions or graphene regions. After layer‐by‐layer printing, the final structure shows the classical airfoil geometry for aerodynamic purpose; its basic PI framework was processed by 0.375 W laser to maximize mechanical strength (12.7 MPa), while three key components were processed by 1 W laser to form smart graphene structures, including leading edge, honeycomb rib, and top/bottom skin. With specific structural design and property control, the graphene components could endow the printed wing with multifunctional performance. Firstly, the smart honeycomb rib (Figure 5c) was designed for recognizing external forces from various directions. The polar coordinate graphing shows clearly that by loading a 0.15 N compressive force from different angles, the resistance change (Δ*R*/*R*_0_) measured by horizontal electrodes varies accordingly depending on the force direction. Specifically, forces from horizontal directions (0° and 180°) introduce minimum Δ*R*/*R*_0_ around 58% and forces from vertical directions (90° and 270°) introduce maximum Δ*R*/*R*_0_ around 105%. Other forces introduce moderate Δ*R*/*R*_0_ depending on the specific angle. Secondly, the smart leading edge (Figure 5d) was designed as a joule‐heating structure with hydrophobic surface for reducing high icing risks. Video S4 in the Supporting Information and snapshot pictures successfully demonstrate the removal of a double‐volume ice block within 7 min by heating the leading edge at 130 °C. Additionally, the surface of the printed graphene shows hydrophobic characteristics with water contact angle of 142.7°, which is also critical for anti‐icing performance by avoiding the trap of cloud/rain droplets. Lastly, the smart skin (Figure 5e) with tunable thickness was designed for high‐temperature resistance (HTR), electromagnetic interference (EMI) shielding, and microwave absorbing. Video S5 in the Supporting Information and snapshot pictures demonstrate the HTR performance by resisting the high‐temperature attack (700–800 °C) of burning fire for at least 6 min. Evaluated by EM S‐parameters, the total EMI shielding effectiveness (SE_Total_) has reached 44.7 dB for the smart skin with 2.5 mm thickness, which exceeds the SE_Total_ of commercial EMI shielding materials (20 dB) and other reported LIG (24.8–32 dB).^[^ ^60^ ^]^ Besides, the smart skin with a nickel treatment has shown much improved reflection losses (RL) with −15.3 dB absorption peak at 9.1 and 2.5 GHz bandwidth (from 8.0 to 10.5 GHz) under −10 dB, representing that 90% of the electromagnetic wave energy has been absorbed. Along with detailed information in Figure S12 (Supporting Information), the performance of SE_Total_ and RL has proved the possibility of LIG‐AM technology for EMI shielding and stealth.

## 3 Conclusion

In summary, we demonstrated a brand‐new LIG‐AM protocol to form bulk 3D graphene with freeform structures without introducing extra binders, templates and catalysts. By triggering both particle‐sintering and graphene‐converting processes layer‐by‐layer, LIG‐AM creatively irradiates PI powder‐bed to assemble varied types of graphene architectures including identical‐section, variable‐section, and unique graphene/PI hybrid structures with random shape complexities. In addition, processing efficiency and assembling resolution of LIG‐AM are also balanceable through synergistic control of lasing power and powder‐feeding thickness. By virtue of outstanding processing advantages, a series of LIG‐AM enabled smart devices/structures, especially the LIG‐AM enabled aircraft‐wing section model, are successfully printed and demonstrated their multifunctional properties to meet the need of potential multiscenario applications.

## 4 Experimental Section

### Fabrication

A laser platform (DLS 2.3, Universal Laser Systems, Inc.) equipped with a CO_2_ laser with wavelength of 10.6 µm and a controlled XYZ‐movement was used for irradiating polymer powders (DuPont Chemical Corp., USA). Typical values of laser power were varied from 0.25 to 5 W with the pulses per inch (PPI) fixed at 500 and the scribing speed at 50 mm s^−1^. The powder spreader (BEVS 1806, BEVS Industrial Co., Ltd.) with the resolution of 10 µm was employed to control each layer thickness. After layer‐by‐layer printing, diverse LIG‐AM enabled graphene architectures were performed under ambient conditions.

### Structural Characterization

SEM was performed with JEOL JSM 7001F at 10 kV to examine the morphologies of various samples. TEM images were taken with a high‐resolution JEOL 2100F operated at 200 kV. Raman spectra were collected with a Horiba HR800 Raman microscope using a 532 nm laser with a power of 5 mW. XRD characterization was performed on Rigaku D/max 2550. XPS measurements were performed with a Thermo ESCALAB 250XI instrument equipped with the energy standard of C1s = 284.8 eV binding energy for charge correction. Thermogravimetric analysis (TGA) was carried out with a NETZSCH STA 449F3 instrument at 10 °C min^−1^ under argon atmosphere. Differential scanning calorimetry (DSC) tests were evaluated with TA Q5000IR instrument at a ramping rate of 10 °C min between 40 and 400 °C. Specific surface area was measured with a Quantachrome 2000e BET surface analyzer.

### Performance Evaluation

Mechanical properties were evaluated through a dynamic mechanical analyzer (DMA, Q850) and an E44.104 tensile machine (MTS Systems). Electrical conductivity was quantified using a Keithley 2450 Sourcemeter. Joule heating temperature was monitored by a FLIR camera with the input DC current provided by a Keithley 2260 power source. Supercapacitive properties were collected by CHI608E electrochemical workstation. Microwave shielding/absorption properties were measured by Vector Network Analyzer (VNA, E5071C) at room temperature. Water contact angles were obtained by water drop angle tester (OCA15 Pro).

### Applications of Smart Devices/Structures

By integrating Ecoflex0030 (Smooth‐On, Inc.) or epoxy resin (Hubei Gemvon New Material Co.), LIG/Ecoflex and LIG/epoxy composites were prepared respectively after the curing process at 60 °C for 6 h. Mechanical‐sensing tests of smart composites (30 × 10 × 2 mm^3^) were carried out by a mechanical–electrical coupling method at a rate of 2 mm min^−1^ and the gauge factor (GF) was calculated by the equation GF = (*R* − *R*_0_)/(*R*_0_ × *ε*), where *R*, *R*_0_, and *ε* are instantaneous resistance, initial resistance, and strain, respectively. Liquid‐sensing tests of 3D graphene sensors (10 × 10 × 2 mm^3^) were applied by monitoring the real‐time resistance change through dripping microvolume acetone from 0.5 to 8 µL. Electrothermal tests of 3D graphene heaters (10 × 10 × 2 mm^3^) were performed by loading electrical input powers ranging from 0.25 to 2 W and deicing test of smart leading edge was conducted by recording the volume change of ice cube with the input power of 1.2 W. 3D graphene based‐supercapacitors (10 × 10 × 2 mm^3^) were fabricated using a three‐electrode system with 1 m H_2_SO_4_ electrolyte, where the counter electrode was a Pt wire and the reference electrode was Ag/AgCl. Adsorption capacity tests of 3D graphene removers for organic solvents and oil were calculated by the ratio between the maximum adsorbed adsorbate quantity M and the original mass m using the balance analyzer. High‐temperature resistance test of smart skin was implemented by observing the structural integrity under an alcohol lamp burning. Ni decorated LIG (LIG/Ni) was obtained by pulse electrodeposition in a standard three‐electrode system controlling the deposition time for 10 min. Electromagnetic waves shielding and absorbing tests of LIG and LIG/Ni microwave absorbers were carried out by waveguide method and coaxial wire method for S‐parameters and EM parameters (complex permittivity and complex permeability), respectively.

## Conflict of Interest

The authors declare no conflict of interest.

## Supporting information

Supporting Information

Supporting Video 1

Supporting Video 2

Supporting Video 3

Supporting Video 4

Supporting Video 5

## Data Availability

Research data are not shared.
