# Supplementary material for: Laser‐Induced Graphene Enabled Additive Manufacturing of Multifunctional 3D Architectures with Freeform Structures
Source: Adv Sci (Weinh). 2022 Nov 27;10(4):2204990. doi: 10.1002/advs.202204990 (PMC9896062; doi:10.1002/advs.202204990)
Supplement: Supplementary file 1 — Supporting Information [file ADVS-10-2204990-s003.pdf]

ADVANCED SCIENCE
Open Access

Supporting Information

for *Adv. Sci.*, DOI 10.1002/advs.202204990

Laser-Induced Graphene Enabled Additive Manufacturing of Multifunctional 3D Architectures with Freeform Structures

*Fu Liu, Yan Gao, Guantao Wang, Dan Wang, Yanan Wang, Meihong He, Xilun Ding, Haibin Duan and Sida Luo**

Supporting Information

Laser-induced graphene enabled additive manufacturing of multifunctional 3D architectures with freeform structures

*Fu Liu, Yan Gao, Guantao Wang, Dan Wang, Yanan Wang, Meihong He, Xilun Ding, Haibin Duan, Sida Luo**

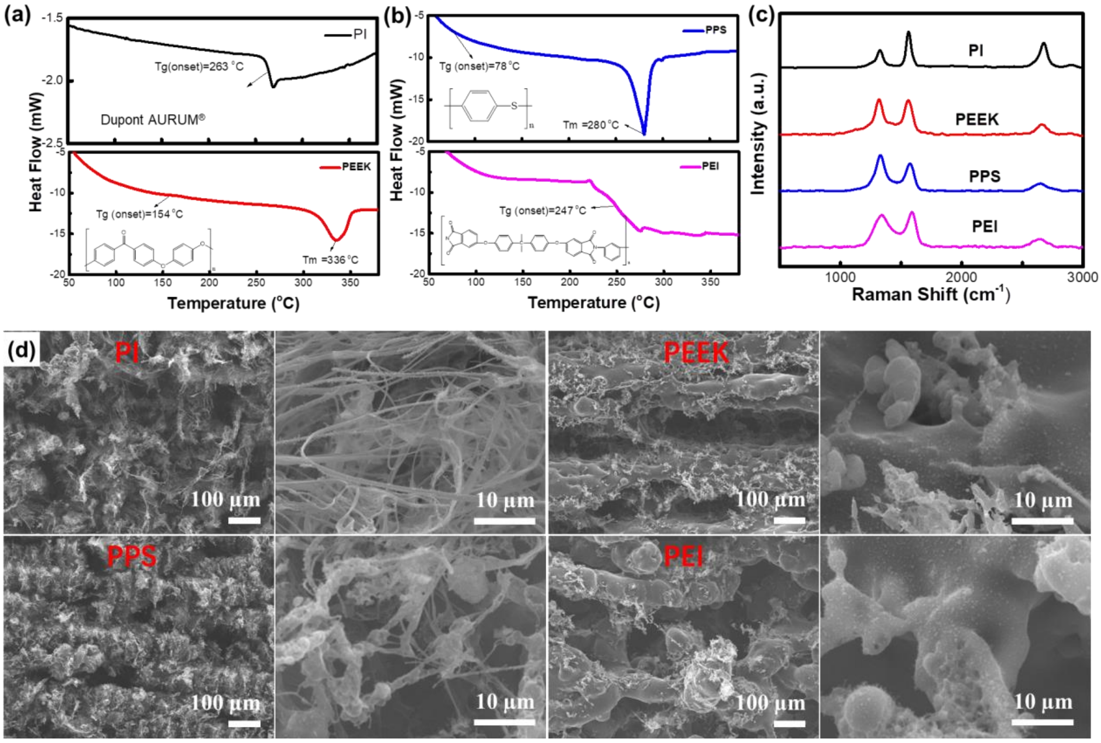

Figure S1. Thermal properties of polymer powders and microstructures of polymer powders derived LIG. a, b) DSC pattern. c) Raman spectra. d) SEM images of LIG structures derived from PI, PPS, PEEK and PEI powders.

Based on the LIG-AM protocol, the thermoplastic polymer is selected according to the fusible performance of the polymer. Typically, most linear thermoplastic polymers with lower melting points tended to molecular chain rupture and gasification rather than yielding LIG during the lasing irradiation process.[1] Thermoplastic polymers containing abundant aromatic groups have been reported to be easily converted into LIG by one-step lasing in ambient atmosphere, suggesting that the aromatic structure was important to the formation of graphene.[2] Therefore, four kinds of thermoplastic polymer powders are included in our investigation scope, namely, thermoplastic polyimide powders (PI), polyether ether ketone powders (PEEK), polyphenylene sulfide powders (PPS), and polyether imide (PEI). DSC tests

were carried out on the four powders respectively. As shown in Figure S1a and b, PI hold the highest glass transition temperature (~263 ℃) suggesting excellent thermal properties due to abundant aromatic groups. Raman spectra for the lased materials shows the three characteristic D, G, and 2D peaks prove that all the four materials can form graphene structures by laser induction, as shown in Figure S1c. The intensity ratio of D peak to G peak (I_D/I_G) of PI-derived LIG was lowest (~0.89) and the intensity ratio of 2D peak to G peak (I_{2D}/I_G) of that was highest (~0.64), indicating the best-quality graphene generation.[3] Furthermore, the distinction of morphology of four polymer powders derived LIG can be observed by SEM (Figure S1d). The surface of PI-derived LIG materials reveals porous graphene structure. Therefore, we preferentially choose PI as the research material among the alternative thermoplastic polymers in this paper.

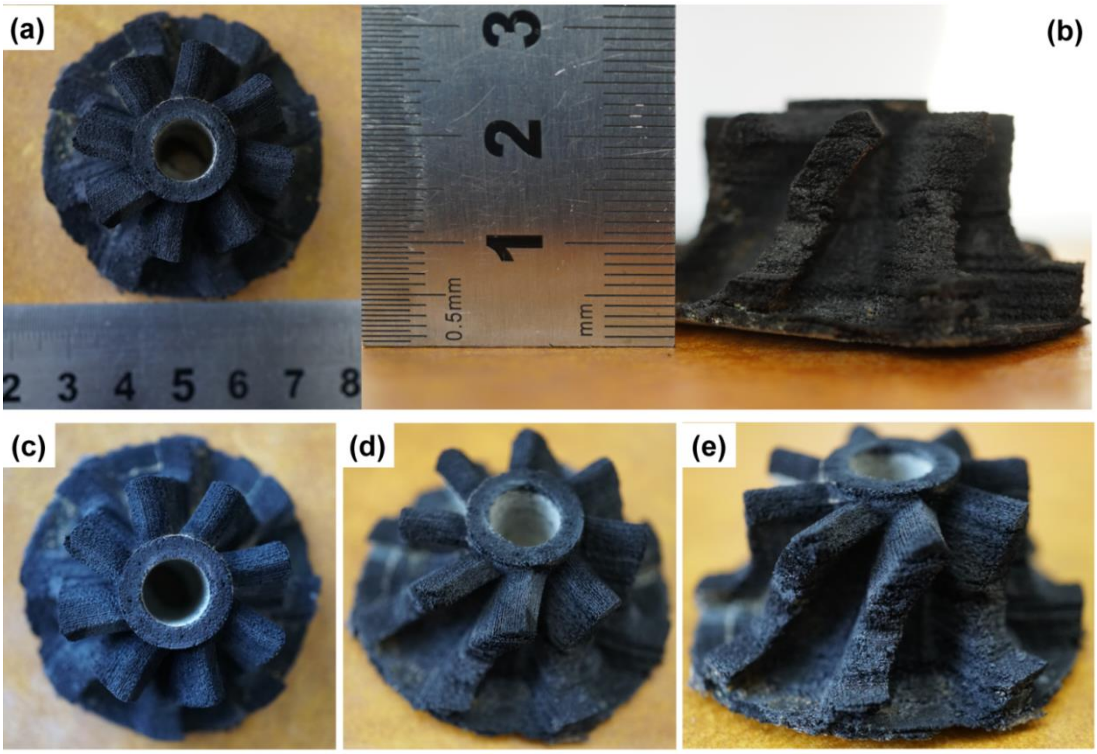

Figure S2. Photographs of the graphene turbine printed by the LIG-AM process. a) The base diameter and b) the height of the sample. c) The top view, d) the side view and e) the front view.

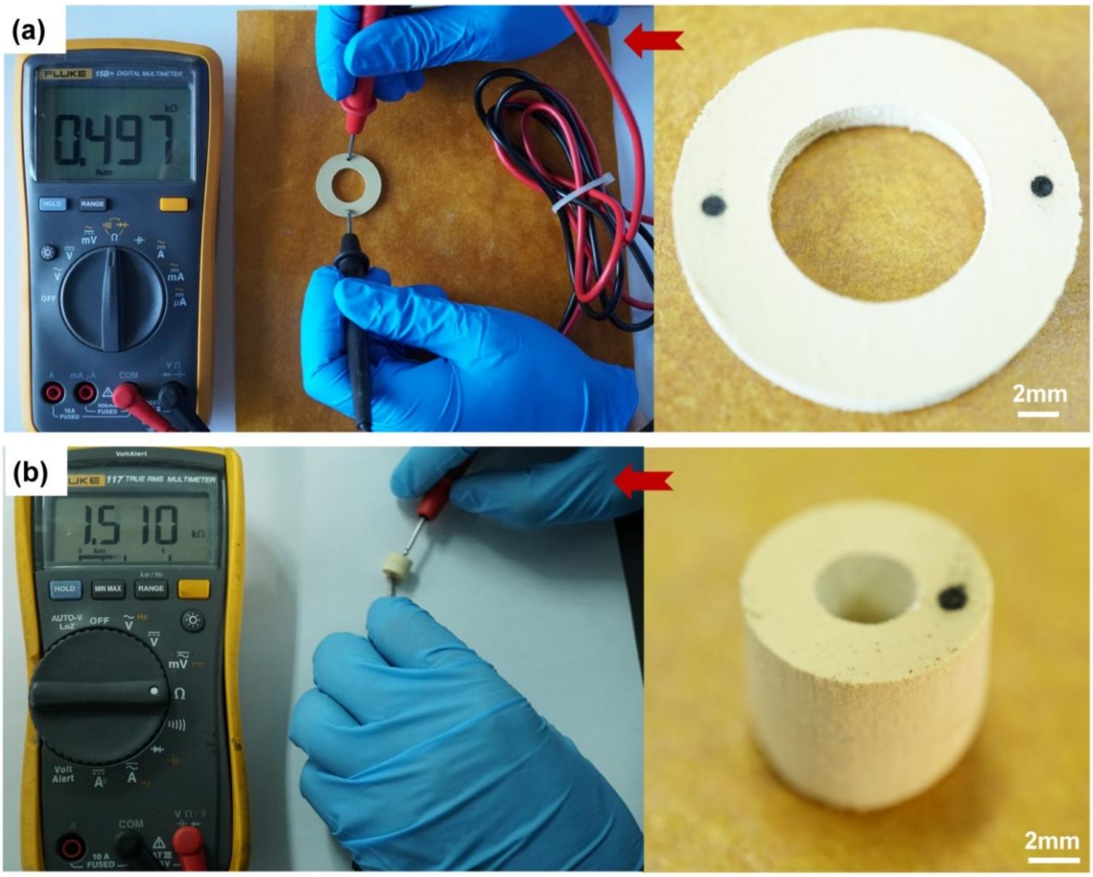

Figure S3. The electroconductibility of the hybrid components. a) The ring component shows a conductive pathway with a resistance of 497 Ω. b) The printed tube shows a resistance of 1.510 kΩ.

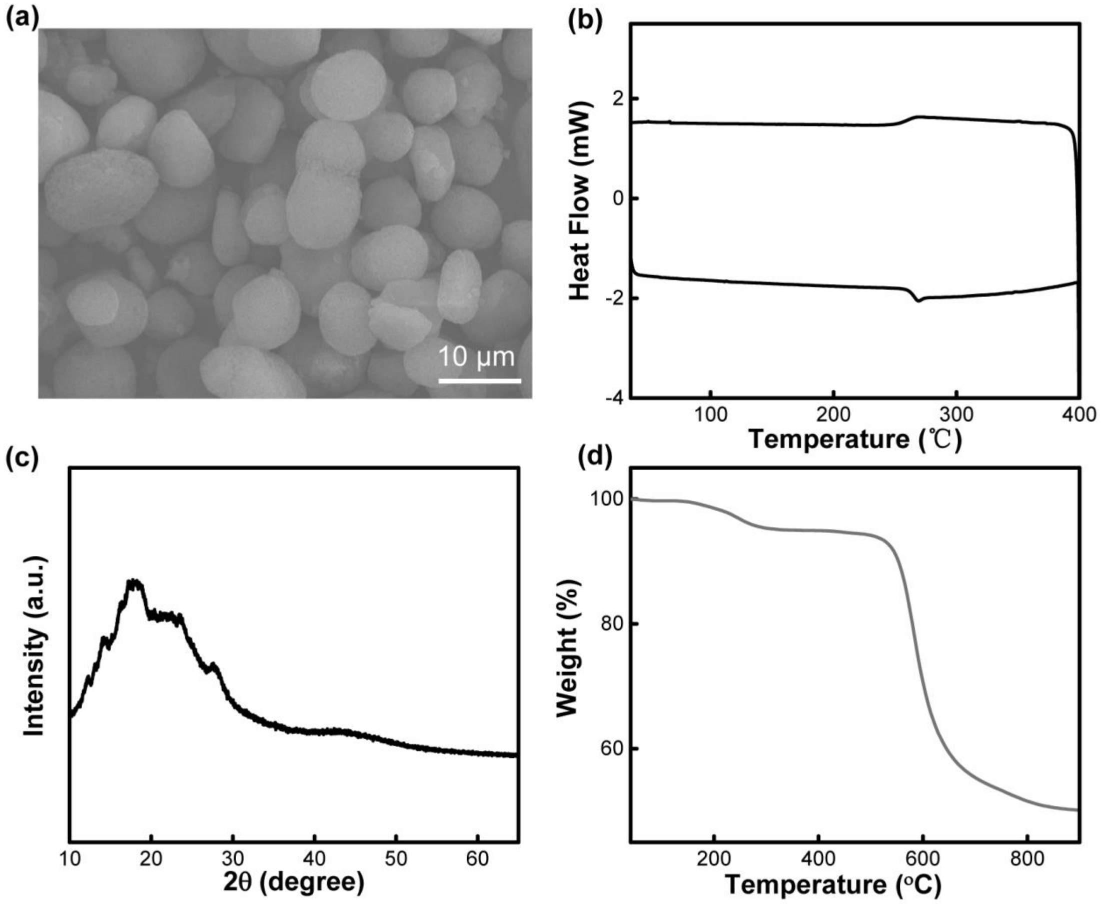

Figure S4. Properties of the polyimide powders. a) SEM image. b) DSC pattern. c) XRD spectra. d) TGA curve in argon atmosphere.

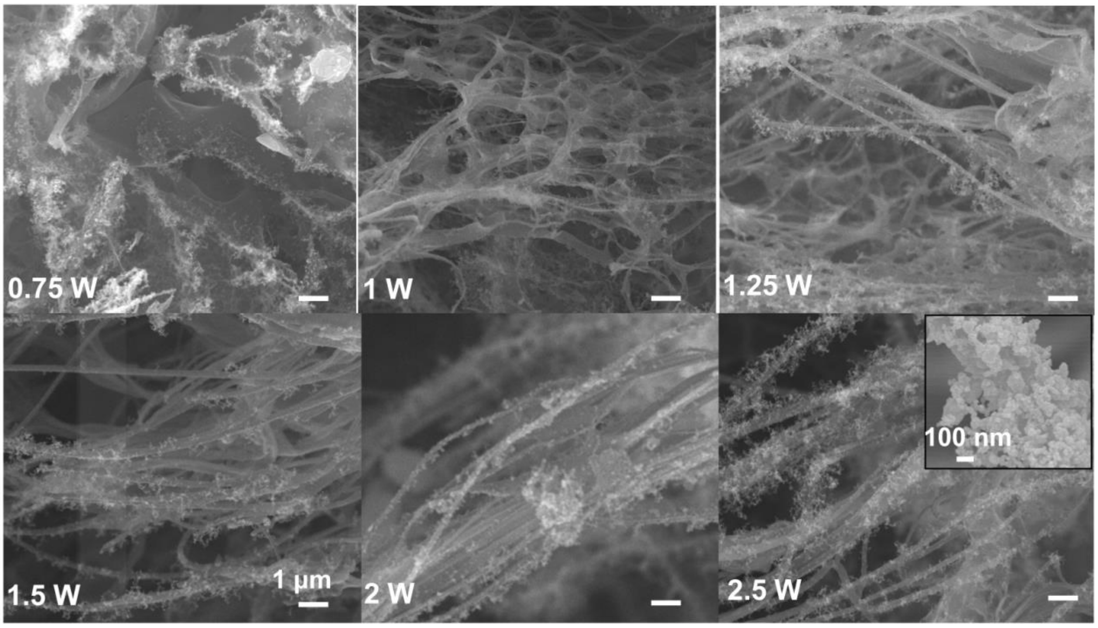

Figure S5. SEM images of LIG structures dependent on laser power. The obtained SEM images describe the dynamic evolution of LIG structures from nanosheets to filamentous fibers with increasing laser power. Compared to the nanosheet structures at 0.75 W, the LIG film obtained at 1 W is full of porous network, which is attributed to the release of gaseous products generated by the massive oxidation under ambient conditions. With further increasing the power to 1.25 W, the porous framework decomposed into fibers with thick foam-like structures. At a high power from 1.5 W to 2.5 W, much shorter fibers attached with carbonized droplets are gradually observed.

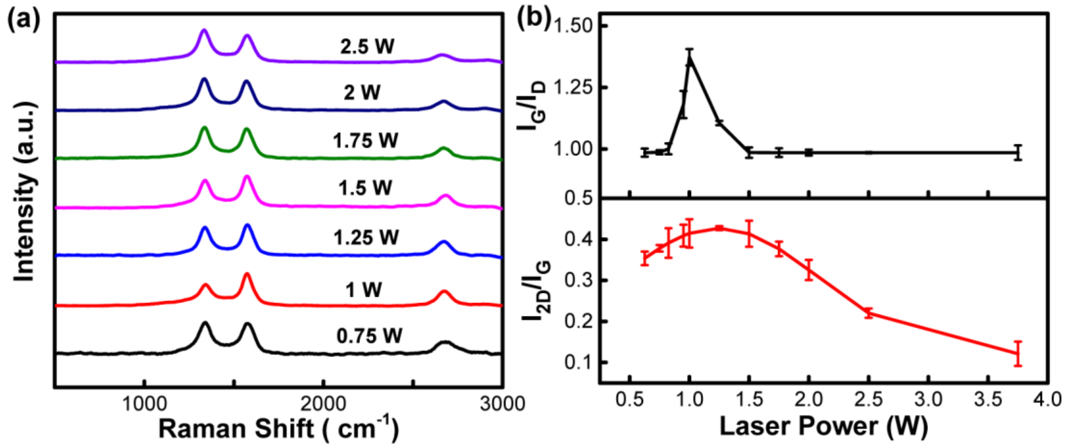

Figure S6. a) Raman spectra of LIG structures induced by various laser powers. b) The dependence of I_G/I_D and I_{2D}/I_G ratios on the laser power.

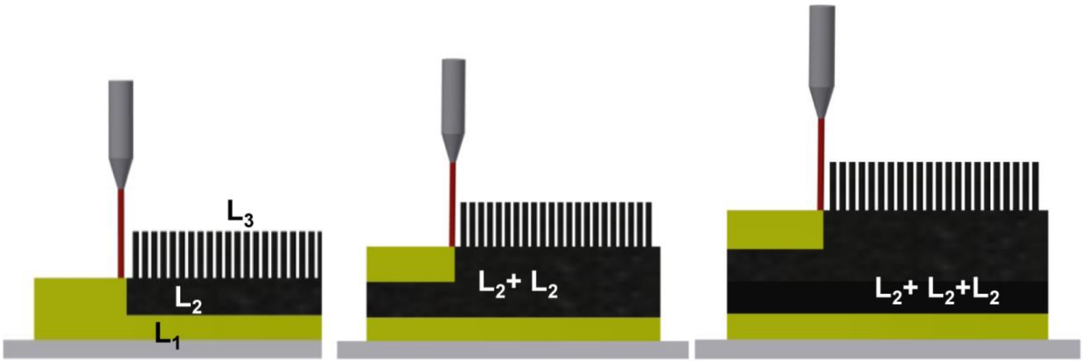

Figure S7. Schematic illustration of the layer-by-layer accumulation of LIG. The dense layer of L_2 plays as a key layer in the printing for multi-layered structures and will accumulate step-by-step as the layering increases. In comparison, the fragile L_3 is vulnerable to damage and will merge with the new feeding layer.

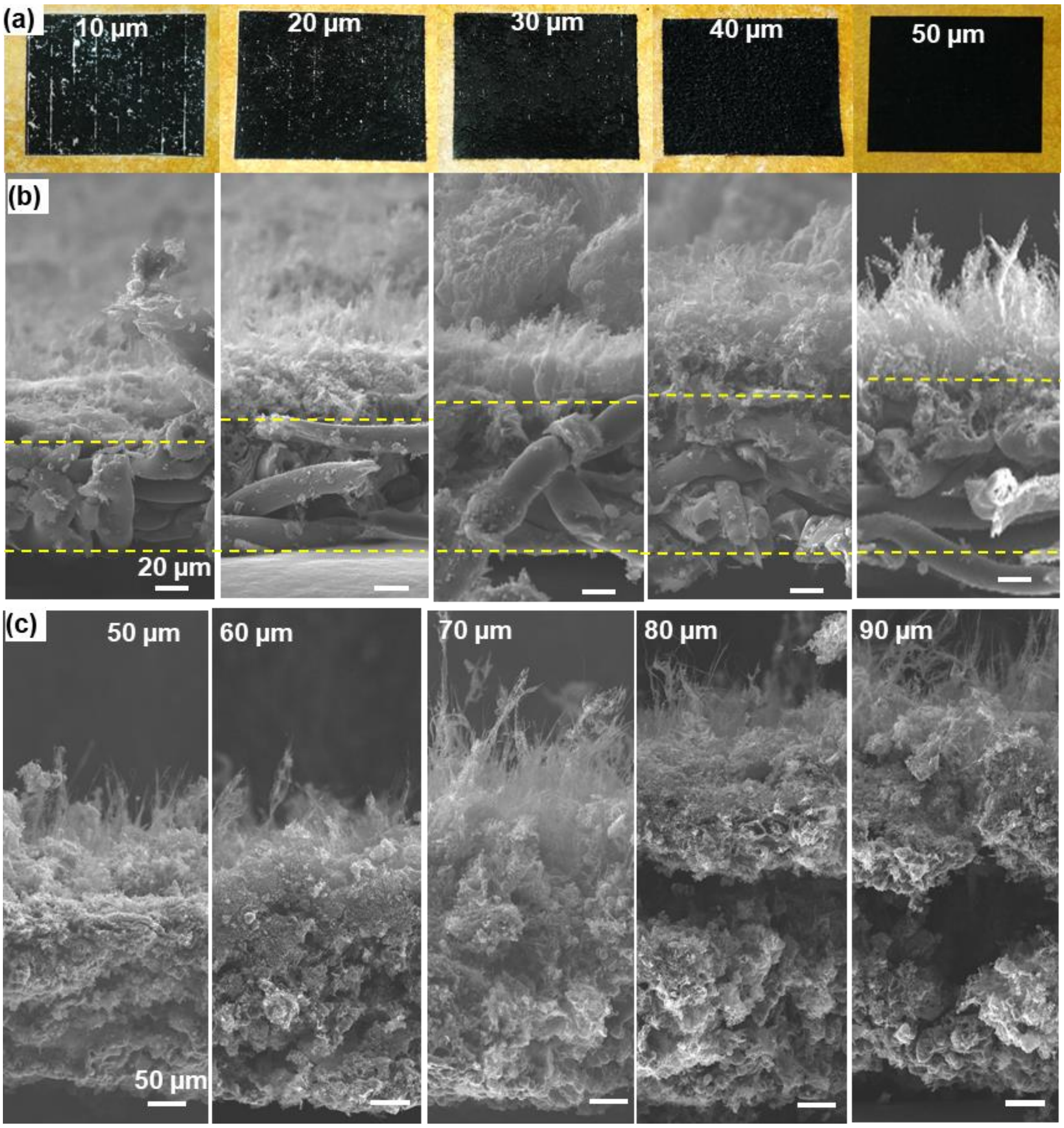

Figure S8. Research of the processing window at 1 W. a) Macrophotographs and b) cross-sectional SEM images of LIG fabricated by varying L_F from 10 μm to 50 μm. It is obvious that when the L_F is less than 40 μm, LIG shows both macroscopic and microscopic defects indicating strong ablation effect. When the L_F > 40 μm, the obtained film maintains the intact structures, suggesting that the available thickness of L_F should be higher than 40 μm. c) Cross-sectional SEM images of 5-layered structures prepared by varying L_F from 50 μm to 90 μm. Within the L_F of 70 μm, uniform multi-layered LIG have been formed. However, when the L_F increases to 80 μm, the obvious delamination emerged due to the loss of interfacial bonding, indicating the maximum L_F should be less than 80 μm. So when L_F lies between ~40 and ~70 μm, the uniform 3D structures can be printed at 1 W.

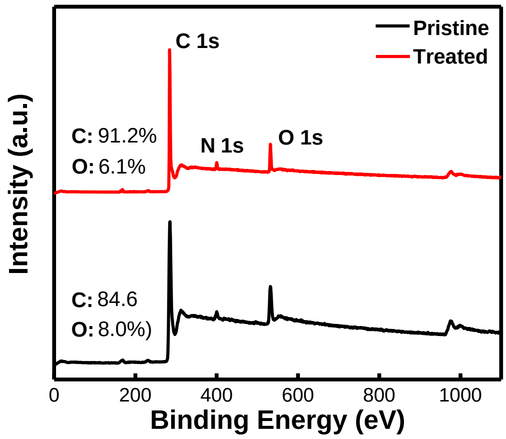

Figure S9. XPS survey spectra of pristine specimen and optimized ones. To improve the printed 3D graphene structure, the DMF solvent (Macklin Reagent Co., Ltd) was utilized to remove the residual powders, followed by a heat treatment at 800 °C to further dissociate the residual impurities. As shown in Figure S9, the C/O ratio increases from 10.6 to 15.0 after the treatment, suggesting an obvious increase in LIG content. The treated samples exhibit excellent porous structures with a low density of 25 mg/cm^3, high specific area of 487 m^2/g, and high porosity of 98.8%, calculated by the equation $\theta = (1 - mV^{-1}d^{-1}) \times 100\%$, where θ, m, V, and d are the porosity, mass, volume, and density of graphite, respectively.[4]

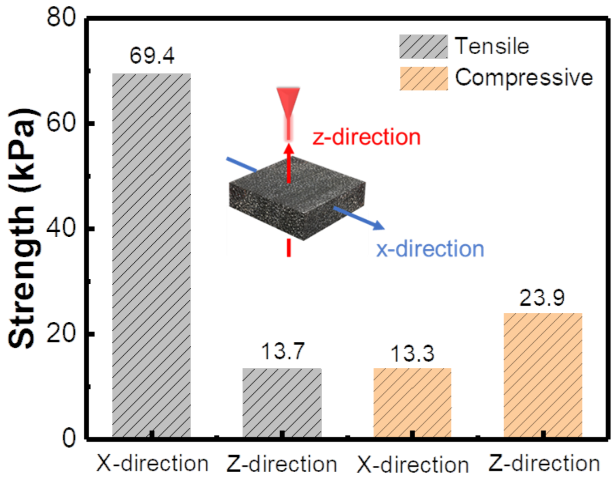

Figure S10. Mechanical strength of 3D graphene structures along different test orientations.

Due to the layer-by-layer process of LIG-AM technique, the construction of 3D-printed graphene is divided into the intra-layered structures and inter-layered structures. To process intra-layered structures, the laser directly processes raw powder-bed to convert PI powders into graphene. To process inter-layered structures, comparatively, the laser not only processes a new powder layer but also bonds the carbonizing PI with the existing graphene structure underneath. Thus, it is reasonable that the processing discrepancy between intra-layered and inter-layered structures could introduce anisotropic mechanical properties at in-plane and through-plane directions. Here, the tensile and compressive strength along both in-plane and through-plane orientations of 3D graphene structures processed under the same conditions have been examined. The inset picture of Figure S10 defines the direction of the laser source perpendicular to the top surface of 3D graphene samples as the Z-direction. Thus, the in-plane properties were tested at X-direction and the through-plane properties were tested at Z-direction. As shown in Figure S10, the in-plane tensile strength 69.4 kPa is larger than the through-plane tensile strength (13.7 kPa). Nevertheless, the in-plane compressive strength (13.3 kPa) is smaller than the through-plane compressive strength (23.1 kPa). It means that the weaker inter-layer bounding strength leads to the anisotropic mechanical properties of the printed specimens along different orientations.

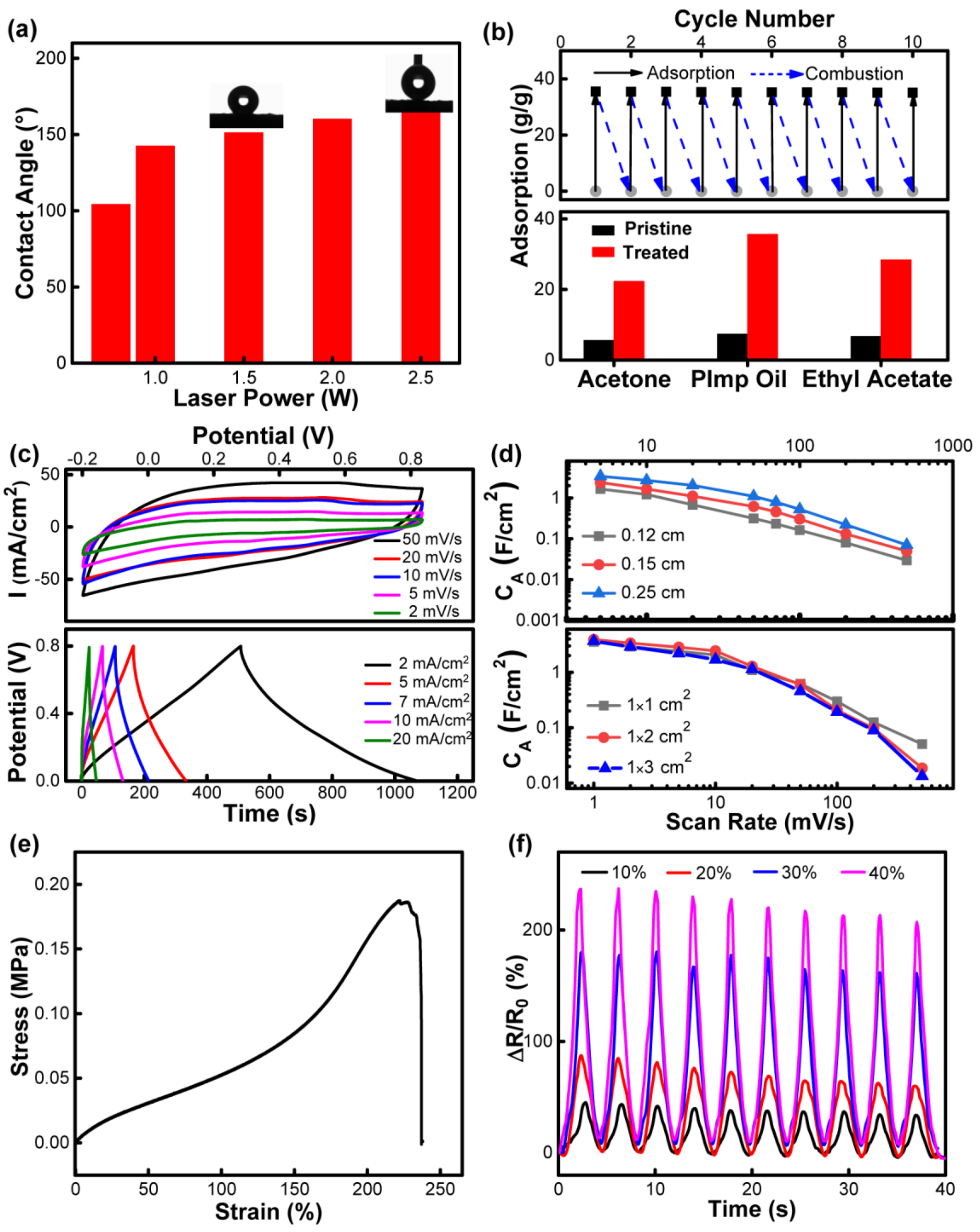

Figure S11. Functional properties of the 3D structures. a) Hydrophobicity. b) Organic solvents and oil adsorption performance. c, d) Supercapacitance performance. e, f) Flexible sensing ability.

The nature of graphene endows the LIG with the hydrophobic which was evaluated by the water contact angles.[5] The summarized water contact angles in Figure S11a demonstrate clearly that the hydrophilicity of 3D structures varies from hydrophobic (104.4°) to super hydrophobic (168.8°) with laser power increasing, due to the evolution of microstructure.[6]

The high porosity enables the 3D structures capacity for acetone, pump oil and ethyl acetate, with approximately 35 times of its own weight, as shown in Figure S11b. Meanwhile, by combustion for removing the adsorbed oil, the recycling adsorption confirm that obtained structures con be reused multiple times without significant loss in adsorption capacity.

The electrochemical test of 3D graphene structures shown in CV curves (Figure S11c, top) exhibit clear pseudo-rectangular shapes, suggesting good capacitive behaviors. The corresponding values of C_A are calculated out ranging from 3.69 to 0.05 F /cm^2 as scan rate from 1 to 50 mV/s. When the voltage scan rate is 1 mV/s, C_V was 24.6 F/cm^3 and C_g is 160.5 F/g. By taking best advantage of the LIG-AM process, 3D-printed graphene with scalable size and shape exhibited excellent and controllable capacitance by expanding the dimensions of 3D graphene structures, which exceed reported 1D graphene fiber,[7] 2D graphene film, [3,8] simple 3D graphene (36.5 F/g),[9] porous rGO foam (81F/g),[10] monolayer 3D graphene structure (100 F/g).[11]. This means that through this AM-LIG process, a simple preparation of ultra-large capacitance capacitors could be achieved by expanding the area or increasing the thickness, indicating high potential of 3D-printed graphene for the field of energy harvesting And CC curves (Figure S11c, bottom) further evidence the well capacitive performance which show the clear near triangle-shaped at varied current densities. It is found that the calculated capacitance from the CC curve at 2 mA/cm^2 (≈1.41 F/cm^2) is approximately equivalent to that from the CV calculated at 10 mV/s (≈1.65 F/cm^2), indicating the validity of the capacitance calculation using the slope of a CC curve.

Serving as both active electrodes and current collectors, free-standing 3D specimens show an increase in C_A by increasing sample thickness from 0.12 to 0.25 cm, which is ranged from 1.65 to 3.41 F/cm^2 at scan rate in 5 mV/s (Figure S11d, top). While the changes of C_V and Cg with the increase of thickness is not obvious at the same scan rate, indicating the homogeneity of 3D samples obtained only changing the number of layers. Moreover, as the area of the work electrodes increase by the same thickness, C_A of different area shows unobvious changes from 1 × 1 cm^2 to 1 × 3 cm^2 (Figure S11d, bottom). This means that through our processing method, we can achieve a simple preparation of ultra-large capacitance capacitors by expanding the area or increasing the thickness, indicating high energy storage capacity for energy harvesting applications. As a demonstration for practical application, three electrolysis cell devices are connected in series to power up LEDs with the thickness of 0.15 cm and working area of 1 × 3 cm^2. As illustrated in supplementary Video S2, 28 red LEDs arrange in the pattern of “BUAA” are lighted over 135 s, showing high energy storage capacity for energy-harvesting devices.

The porous structure of 3D graphene enables it to be integrated with polymers for fabricating functional composites. Figure S11e demonstrates the mechanical-electrical performance of LIG/Ecoflex, showing a large tensile strain of 230%. Excellent integrity in contour and significant improvement in mechanical behavior of LIG-based composites indicated the efficient synergistic output of both the polymers and LIG. Figure S11f further shows the variation in relative resistance ($\Delta R/R_0$) of cyclic stretching-releasing strains at 10%, 20%, 30%, and 40%, and the corresponding values are 34%, 64%, 160% and 210%. The role of 3D graphene in flexible and sensitive composites endows strain sensors with the high sensitivity and large workable range.

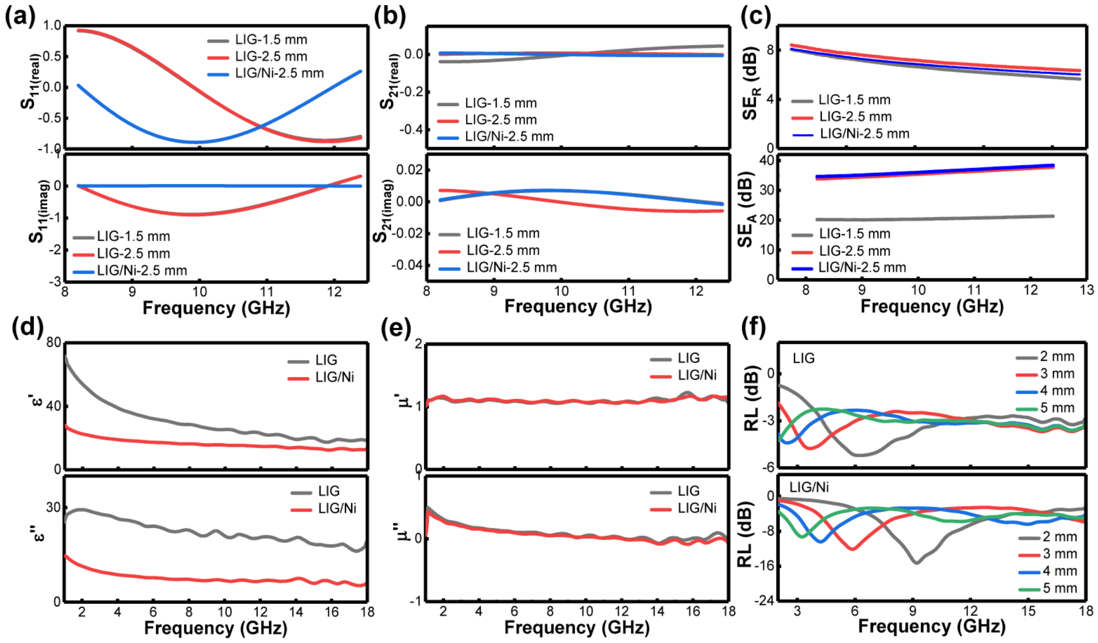

Figure S12. a, b) S-parameters, c) SE_A and SE_R of different LIG and LIG/Ni samples in the X-band. d) Complex permittivity, e) complex permeability, and f) reflection losses (RL) for LIG and LIG/Ni paraffin wax composite samples in the frequency range of 2 – 18 GHz.

Owing to the excellent conductivity performance of 3D graphene foam, the total EMI shielding effectiveness (SE_{Total}) of LIG-1.5 mm reaches up to 28.3 dB (Figure 5e) with the reflection EMI shielding effectiveness (SE_R) of 8.1 dB (Figure S11c), while SE_{Total} for commercial application is only 20 dB.[12] According to the electromagnetic theory,[13] the thickness of the shield is positively correlated with the absorption EMI shielding effectiveness (SE_A) including the contribution of multiple reflections to enhance EMI shielding effectiveness. To prove it, LIG-2.5 mm is prepared to compare the EMI shielding effectiveness with LIG-1.5 mm. As shown in Figure 5e, LIG-2.5 mm has been proved to hold the higher SE_{Total} than that of LIG-1.5 mm, reaching 44.7 dB, which exceed the EMI SE of commercial standard[12] and reported laser induced graphene (24.8 dB,[14] 25.1 dB,[15] 32 dB[16] at 8.2 – 12.4 GHz). It means that the superior EMI shielding performance of 3D graphene can be further enhanced by increasing its thickness which shows great potential in the field of electromagnetic shielding. Figure 5e shows the calculated RL results of the LIG and LIG/Ni for the assumed thickness of 2 mm and Figure S12f shows that of the LIG and LIG/Ni with various thicknesses. Surprisingly, compared with LIG, the obtained LIG/Ni exhibits a breakthrough in the improvement of microwave absorption. With a thickness of 2 mm, the optimal absorption peak reached – 15.3 dB at 9.1 GHz, and the bandwidth corresponding to RL at – 10 dB is 2.5 GHz (from 8.0 to 10.5 GHz). Generally speaking, the

frequency range of RL ≤ – 10 dB is referred to as the effective absorption bandwidth (EAB) where RL = −10 dB represents that 90 % of the electromagnetic wave energy has been absorbed. There are two possible reasons for this phenomenon. On the one hand, the introduction of trace Ni particles provides magnetic loss. On the other hand, the effective connection between the nickel particles and graphene performs an improved impedance matching capability, thereby effectively improving the electromagnetic wave absorption efficiency.[17-19] It means that the superior RL performance of LIG can be further enhanced by increasing doping content of nickel particles which shows great potential in the field of microwave absorption.

Reference

[1] Y. Chyan, R. Q. Ye, Y. L. Li, S. P. Singh, C. J. Arnusch, J. M. Tour, *ACS Nano* **2018**, *12*, 2176.

[2] R. Q. Ye, Y. Chyan, J. B. Zhang, Y. L. Li, X. Han, C. Kittrell, J. M. Tour, *Adv. Mater.* **2017**, *29*, 1702211.

[3] J. Lin, Z. W. Peng, Y. Y. Liu, F. Ruiz-Zepeda, R. Q. Ye, E. L. G. Samuel, M. J. Yacaman, B. I. Yakobson, J. M. Tour, *Nat. Commun.* **2014**, *5*, 1.

[4] J. Sha, Y. Li, R. V. Salvatierra, T. Wang, P. Dong, Y. Ji, S. Lee, C. Zhang, J. Zhang, R. H. Smith, P. M. Ajayan, J. Lou, N. Zhao, J. M. Tour, *ACS Nano* **2017**, *11*, 6860.

[5] O. Leenaerts, B. Partoens, F. M. Peeters, *Phys. Rev. B* **2009**, *79*, 235440.

[6] Y. Wang, G. Wang, M. He, F. Liu, M. Han, T. Tang, S. Luo, *Small* **2021**, *17*, 2103322.

[7] T. Huang, B. Zheng, L. Kou, K. Gopalsamy, Z. Xu, C. Gao, Y. N. Meng, Z. X. Wei, *RSC Adv.* **2013**, *3*, 23957.

[8] Z. S. Wu, K. Parvez, X. Feng, K. Müllen, *Nat. Commun.* **2013**, *4*, 2487.

[9] Z.Y. Niu, Y. Zhang, Y. Zhang, X.Z. Lu, J.H. Liu, *J. Alloys Compd.* **2020**, *820*, 153114.

[10] T. Purkait, G. Singh, D. Kumar, M. Singh, R.S. Dey, *Sci. Rep.* **2018**, *8*, 640.

[11] S. Drieschner, M. Weber, J. Wohlketzetter, J. Vieten, E. Makrygiannis, B.M. Blaschke, V. Morandi, L. Colombo, F. Bonaccorso, J.A. Garrido, *2D Mater.* **2016**, *3*, 045013.

[12] Z. P. Chen, C. Xu, C. Q. Ma, W. C. Ren, H. M. Cheng, *Adv. Mater.* **2013**, *25*, 1296.

[13] H. L. Lv, Y. H. Guo, G. L. Wu, G. B. Ji, Y. Zhao, Z. C. J. Xu, *ACS Appl. Mater. Interfaces* **2017**, *9*, 5660.

[14] W. J. Yu, Y. Y. Peng, L. J. Cao, W. W. Zhao, X. Q. Liu, *Carbon* **2021**, *183*, 600.

[15] X. Han, R. Q. Ye, Y. Chyan, T. Wang, C. H. Zhang, L. L. Shi, T. Zhang, Y. Zhao, J. M. Tour, *ACS Appl. Mater. Interfaces* **2018**, *1*, 5053.

[16] J. Yin, J. X. Zhang, S. D. Zhang, C. Liu, X. L. Yu, L. Q. Chen, Y. P. Song, S. A. Han, M. Xi, C. L. Zhang, N. Li, Z. Y. Wang, *Chem. Eng. J.* **2021**, *421*, 129763.

[17] Y. L. Zhang, X. X. Wang, M. S. Cao, *Nano Res.* **2018**, *11*, 1426.

[18] X. X. Wang, T. Ma, J. C. Shu, M. S. Cao, *Chem. Eng. J.* **2018**, *332*, 321.

[19] N. Li, C. W. Hu, M. H. Cao, *Phys. Chem. Chem. Phys.* **2013**, *15*, 7685.
